# A Comparison of 3 Ways of Conventional Pap Smear, Liquid-Based Cytology and Colposcopy vs Cervical Biopsy for Early Diagnosis of Premalignant Lesions or Cervical Cancer in Women with Abnormal Conventional Pap Test

**Published:** 2013-12

**Authors:** Mojgan Karimi-Zarchi, Fateme Peighmbari, Neda Karimi, Mitra Rohi, Zohre Chiti

**Affiliations:** Shahid Sadoughi University of Medical Science, Yazd, Iran

**Keywords:** cervico-vaginal cytology, liquid- base cytology, conventional pap, atypical squamous cell, colposcopy, biopsy

## Abstract

The most cost effective method of prevention and detection of cervical cancer is the Pap smear. In abnormal Pap smear, colposcopy, endocervical curettage and biopsy will be done. Gold standard method in detecting cervical lesion is biopsy. Now in two ways conventional Pap smear and liquid base are routine diagnostic technique in Iran and given easily and cost-effectiveness of this method in the detection of cervical lesions to determine the sensitivity the objective of this study was compare three methods of Pap smear and colposcopy in detection of any lesion to gold standard biopsy in the positive ASC cases who referred to gynecologic Oncology Clinic of shahid Sadoughi University of Medical Science. This study is a descriptive analytic in 2009-2010 years on 150 cases of patients with Atypical Squamose Cell (ASC) results in previous pap smear ,conventional pap smear, liquid based pap smear, colposcopy and cervical biopsy had been done for all patient and finally data were analyzed with chi-square statistical test on spss ver 16 saftware. Average age of patients in this study was, 42 ± 9.9 year and reason for referring patients in 35.4% of cases was due to follow-up of abnormal results of previous Pap smear, in 30% bleeding, 12% Pain and 2.6% percent of cases was checking-up. In final results of sensitivity, specificity, positive predictive value (PPV), negative predictive value (NPV) and accuracy any of the methods conventional and liquid based Pap smear and colposcopy were compared with cervical biopsy as a gold standard. The conventional Pap smear method had a sensitivity 51%, specificity 66.6%, PPV 96%, NPV was 8% and accuracy was 92%, about the liquid base Pap smear method, sensitivity was 55.3%, specificity was 77.7%, PPV was 97.5%, NPV was 10% and accuracy was 56/6%. About the colposcopy, sensitivity was70/9 % specificity 44/4%, PPV was 95.2%, NPV was 8/8% and accuracy was 69.3%. The relationship between sensitivity results of conventional Pap smear and colposcopy, with *p*<0.001 and between the results of sensitivity of liquid based cytology and colposcopy with *p*<0.01 relationship was significant. Colposcopy has the best efficacy in detecting any cervical lesion in compared with any other diagnostic technique. so that further studies with more detailed plans and bigger sample sizes are suggested for obtaining reliable result.

## INTRODUCTION

The most cost effective method for the prevention and detection of cervical cancer is the pap smear test, and when the test result is abnormal, colposcopy and endocervical curettage or biopsy may be necessary to perform. However, the definite method to diagnose cervical cancer is biopsy (1, 7, 11, 13).

Recent studies in developing countries have shown that in cases of abnormal pap smear results of ASC, using a relatively new method of Liquid-based cytology (LBC) and colposcopy are more helpful in comparison with conventional pap smear (2, 5, 8, 16) since it requires fewer tests which reduces further visits to the clinic (12).

Given the fact that this evaluation has not been carried out in our country, needs for comparing three screening methods in dealing with abnormal pap ASC is an important point to consider.

Since the results of ASC in pap smear are common in our country and there is no accurate way to evaluation, this causes the need for several visits and invasive procedures such as unnecessary biopsy which themselves cause tremendous cost and unwanted complications for patients. Therefore, if an alternative way than conventional pap smear could be found it would help the patients significantly. LBC method has been the choice from past 8 years in developed countries that still have not been able to find a suitable place in our country (9). The aim of this study was, therefore, to compare three methods of conventional pap smear, LBC, and colposcopy on ASC pap smear results hoping by introducing the colposcopy or LBC methods as a superior and cost-effective methods reducing the biopsy requirements and also the need for future and frequent visits.

## METHODS

In this study, 150 patients with previous pap smear result of ASC, who visited the oncology clinic of The Shahid Sadoughi Hospital, underwent conventional pap test and LBC. At the same session, coloposcopy as well as biopsy was performed on these patients. The result of each patient was compared with other patients and elements such as: sensitivity, specificity, positive predictive value, and negative predictive value were also addressed. The code was given to each of these reviews to make sure that pathologist reads and records the results of pap smear, LBC, colposcopy, and biopsy without any knowledge of the patient’s condition. It should be noted that patient's consent was taken before they entering the study and before any action was to be taken. Collected data, using a questionnaire, was poured into the computer software SPSS ver. 16 and from that the tables and indexes were obtained. Chi-square and z-test were also used for statistical comparisons.

## RESULTS

In this study, 150 patients were studied with average age of 42 ± 9.94 years, and range of 21 to 70 years of age. Eighteen (12%) of the patients came to the clinic with pain, 46 patients (30.7) with bleeding and 29 patients (19.3) with a complaint of vaginal discharge. Four patients (2.6%), came to the clinic for check up, and 4 (35%) for following up their previous results. All 150 patients (100%) had ASC results in their previous pap smears.

All patients were reviewed for having a family history of breast cancer, ovarian cancer and endometrial cancer in which 29 patients (19.3%) were found to have a positive family history with nine of them (6%) having breast and endometrial cancer, two of them (1.3%) having ovarian cancer, and 9 patients (6%) having other cancers including: colon, skin, etc. Sixty four patients (42.7%) were premenopausal and 86 still had their menstrual periods. Efficacy of the samples was about 100%.

On assessment of the distribution of the relative frequency of ASCUS, 27 (18%) cases of ASCUS were reported on conventional pap smear, 19 (13.7%) on LBC pap smear, and none on colposcopy and biopsy.

The relative frequency of ASC-H was found on 3 (2%) patients in conventional pap smear, 9 (7%) patients in LBC, and none in colposcopy and biopsy technique. Also the relative incidence of cervicitis were found in 22 patients (14.7%) in conventional pap smear, 6 patients (4%) in pap smear LBC, 44 patients (28.7%) in colposcopy, and 89 (59.3%) in biopsy (Table 1).

**Table 1:** The comparison of premalignant lesion in pap versus cervical biopsy

Biopsy	Distribution of the conventional pap in HSIL detection compared with biopsy						Distribution of the conventional pap in LSIL detection compared with biopsy					
	NO		YES		ALL		NO		YES		ALL	
Conventional Pap	percent	number	percent	number	percent	number	percent	number	percent	number	percent	number

NO	88.7	133	5.3	8	94	141	82.7	124	9.3	14	92	138
YES	5.3	8	0.7	1	6	9	6.7	10	1.3	2	8	12
ALL	94	141	6	9	100	150	89.4	134	10.6	16	100	150

According to the table, comparing the frequency of LSIL between conventional pap smear and biopsy, sensitivity of 12.5% and positive predictive value of 16.6% were reported. Specificity of conventional pap smear and negative predictive value in diagnosing of LSIL were 92.5% and 89.8% and compliance was 84%. A *p* value of 0.483 was obtained in Chi-square test which shows no relationship between the conventional pap smear and biopsy in the diagnosis of LSIL. Comparing the frequency of HSIL on conventional pap smear and biopsy, specificity above 94.3% and a sensitivity of 11.1% and negative predictive value of 94.3%, and positive predictive value of 11.1% and compliance of 89.3%, were found. Chi-square test showed p value of 0.505 which shows there is no relationship between the conventional pap smear and biopsy in the diagnosis of HSIL.

Table 2 compares the frequency of LSIL in LBC pap smear with biopsy and shows a specificity of LBC 7.94%, sensitivity of LBC 18.7%, and negative predictive value of 90.7%. Positive predictive value was 30% and compliance was about 86.6%. Chi-square test shows a p value of 0.400 which means there is a significant relationship between LBC and biopsy in the diagnosis of LSIL. Comparing the frequency of HSIL in LBC pap smear with biopsy, a specificity of LBC 75.8%, sensitivity of 66.6%, negative predictive value of 97.2%, positive predictive value of 15%, and compliance of 75.3% were obtained. According to the Chi-square test (*p* value=0.011) a significant relationship was observed between LBC and biopsy in the diagnosis of HSIL.

**Table 2:** The comparison of lesions in liquid-based cytology versus biopsy

Biopsy	Distribution of the LBC pap in HSIL detection compared with biopsy						Distribution of the LBC pap in LSIL detection compared with biopsy					
	NO		YES		ALL		NO		YES		ALL	
LBC Pap	percent	number	percent	number	percent	number	percent	number	percent	number	percent	number

NO	71.3	107	2	3	73.3	110	84.6	127	8.7	13	93.3	140
YES	22.7	34	4	6	26.7	40	4.7	7	2	3	6.7	10
ALL	94	141	6	9	100	150	89.3	134	10.7	16	100	150

Table 3 compares the frequency of LSIL in colposcopy with biopsy and shows a specificity of 67.1%, sensitivity of 56.2%, negative predictive value of 92.7%, positive predictive value of 16.9%, and compliance of 66%. Using Chi-square test, *p* value of 0.064 obtained which shows there is no relationship between colposcopy and biopsy in the diagnosis of LSIL. Comparing the frequency of HSIL between colposcopy and biopsy, a specificity of 97.8, a negative predictive value of 93.8, sensitivity and positive predictive value of zero, and compliance of about 92% was obtained. Chi-square test (*p* value=0.658) showed no significant relationship between colposcopy and biopsy in the diagnosis of HSIL.

**Table 3:** The comparison of lesions in colposcopy versus biopsy

Biopsy	Distribution of the colposcopy in HSIL detection compared with biopsy						Distribution of the colposcopy in LSIL detection compared with biopsy					
	NO		YES		ALL		NO		YES		ALL	
Colposcopy	percent	number	percent	number	percent	number	percent	number	percent	number	percent	number

NO	92	138	6	9	98	147	60	90	4.7	7	64.7	97
YES	2	3	0	0	2	3	29.3	44	6	9	35.3	53
ALL	94	141	6	9	100	150	89.3	134	10.7	16	100	150

Finally three methods of conventional pap smear, LBC pap smear, and colposcopy to identify any cervical lesion were compared using a z-test and the following results were obtained:

1. In evaluation of the sensitivity of two methods, conventional pap smear and LBC pap smear, using Chi-square, p value of <0.05 were reported which means there is no significant relationship between these two methods and it is not sensible to compare the sensitivity of these two methods.
2. Using Chi-square, the sensitivity of the conventional pap smear and colposcopy were compared with p value of 0.001which demonstrates a significant relationship between these two methods. colposcopy procedure described in this study with 70.6% sensitivity was superior comparing to conventional pap with sensitivity of 51%.
3. The sensitivity of LBC pap smear and colposcopy were compare using Chi-square test and *p* value of <0.01 was reported which shows a significant relationship between these two methods to diagnose any cervical lesions. Colposcopy with sensitivity of 70.9% can be stated to be superior than LBC pap with sensitivity of 55.3%.
4. The specificity of three above procedures using a Chi-square test was evaluated and a *p* value of *p*>0.05 shows no correlation between these three methods.
5. The positive predictive value and negative predictive value were also compared using Chi-square test (*p*<0.05) and a significant relationship was noted.
6. The compliance of conventional pap smear was compared with that one of colposcopy and a significant relationship was found (Chi-square, *p* value>0.01) which means colposcopy with compliance of 69.3% is superior to the conventional pap with compliance of 52%.
7. In the comparison of compliance of the conventional pap smear with that one of the LBC no significant relationship was found (Chi-square, *p* value<0.05).
8. Chi-square test (*p* value=0.022) indicates that there is a significant correlation between LBC pap smear and colposcopy which means colposcopy with compliance of 69.3% is superior to the LBC pap with compliance of 52%.

## DISCUSSION

Cervical cancer is the most common cancer in women and about 500 thousand women annually in the developing world lose their lives to this cancer. Today, with proper implementation of the pap smear many cases of invasive disease has been prevented.

Our goal in the current study was to compare three methods of conventional pap smear, LBC pap smear, and colposcopy in cases with ASC result in order to introduce the LBC or colposcopy procedures as the preferred and more economical method to reduce the need for future visit and or patient biopsy.

Previous related assessments and reviews which compared three methods, conventional pap smear, LBC and colposcopy, somewhat found that LBC and colposcopy are better than conventional pap smear at detecting pre-cancerous lesions.

As in similar studies in 2003 by annie and colleagues found that LBC method was superior to detect SIL lesions (5.1% vs. 3.5%) especially HSIL lesions compared to the conventional pap smear (1% vs. 0.5 %) (13). They also reported that detecting the ASCUS and LSIL lesions was lower in LBC than the conventional pap smear and LBC demonstrated no change in detecting the lesions of SCC, adenocarcinoma and HSIL compared to the conventional pap smear method (13).

Also in another study reviewing the ASCUS cases, it was reported that conventional pap smear was 18% accurate and LBC pap smear was 13.7% which shows a reduction in cases of ASCUS in LBC method. This was in agreement with the results of Annie and colleagues in 2003 (13). Finally, in a general and comprehensive review, the sensitivity, specificity, positive predictive value, negative predictive value, and the compliance of three methods: conventional pap smears, LBC and colposcopy, in diagnosis of any cervical lesions was determined based on biopsy results. The table presented in this study shows that the sensitivity of the conventional pap smear compared to the LBC did not reached significance. The sensitivity of the conventional pap smear 51%, colposcopy 70.9%, and sensitivity of LBC 55.3% which reached significant level and shows a higher sensitivity of the colposcopy procedure in detecting any cervical lesions in this study. Unfortunately, the specificity and positive predictive value and negative predictive value did not show any significant relationship but compliance of the colposcopy procedure (69.3%) was higher compared to the conventional pap smear (52%) and LBC (56.6%) [similar to a 2001 study by Stuart and colleagues in which they showed that colposcopy was more consistent with biopsy than the other two methods (8)].

In a study done by John and his colleagues in 2002 at Mary Hospital in Hong Kong, two methods; conventional pap smear and LBC, in detecting squamous intraepithelial lesions (SIL) were compared. In this study, SIL lesions detected by pap smear LBC 5.1% and SIL lesions detected by conventional pap 3.5% reported, which among those HSIL lesions detected by LBC was 1% and by conventional pap smear was 0.5%. Also detection of ASUS and AGUS lesions was higher by LBC method than conventional pap smear, which it suggests that using LBC for detecting the intraepithelial lesions and cervical screening is advanced than using the conventional pap smear (14).

As noted earlier, in similar studies in 2009 by Beermana and colleagues discontent with samples was decreased in a LBC pap smear and the detection of ASCUS and sensitivity of LBC in detecting the cervical cytology lesions was higher (while in the current study, diagnosis of ASCUS using LBC method was lower compared with conventional pap smear) (7).

In 2004, a study conducted by Schlderman and colleagues at the Denmark Hospital, a reduction in the incidence of atopia in the cervical cytology specimens using LBC pap smear was reported but the incidence of dysplasia from mild to severe were higher in LBC compared to the conventional pap smear (1).

In a study conducted in 2008 by syrjanen on the comparison of the two LBC and conventional pap smear methods with standard diagnostic colposcopy, in general, they concluded that the LBC has a higher sensitivity, but due to the nature of the study that they divided the patients into two groups: under 35 and over 35 years old, they concluded that for people over 35 years old LBC method is better than the conventional pap smear method [2008]. But in this study due to the low number of samples and the fact that majority of the patients were older than 35 years of age, this separation did not performed (5).

In a study conducted in 2002 by Apgar and her colleagues, comparison of three methods of conventional pap smear, colposcopy and HPV was performed and the HPV was found to be the best. In this study, HPV testing did not performed (3).

In a study done in 2010 by Doctor Karimi and her colleagues comparing the colposcopy and conventional pap smear with biopsy standard diagnostic showed that the colposcoy method has higher sensitivity and specificity compared to the conventional pap smear (16). In another study performed by Doctor Karimi and her colleagues in 2011, sensitivity and specificity of repeated pap smears for ASCUS was reported to be 15% and 93%, respectively, while the sensitivity and specificity of colposcopy in diagnosis of cervical cancer was 80% and 80%, respectively. Considering the fact that the low accuracy of pop smear in Iran as a developing country and the need for early detection of cervical cancer, cervical biopsy and colposcopy is recommended for patients (10).

Setsu akamatsu and his colleagues in a study published in 2012 concluded that LBC technique has significantly lower percentage of unsatisfactory samples than conventional method (respectively, 1.38 and 11.45% and *p*<0.01). Among women in primary tumor lesions in 0.57 % of those who were evaluated using LBC, identification were significantly higher than the percentage of reported positive in those of the conventional method (*p*<0.05, 0.25). Repeated screening among women, disease was detected in 0.08% of women who were reviewed using LBC which significantly was less than the percentage of those that were positive in the conventional method following the LBC (0.11%) or were re-examined by conventional method (0.16 and *p*<0.05).

LBC method was reported to be significantly more than conventional method at a low enough rate and the rate of high diagnosis of cancer, cervical cancer screening in an area of Japan (3, 6, 10, 14, 15).

## CONCLUSION

The results of this study demonstrate that in general colposcopy method has a higher sensitivity in diagnosis of any cervical lesions compared to the conventional pap smear and liquid-based pap smear and also statistically significant relationship was found between them. However, in the comparison of the conventional pap smear and liquid-based pap smear in terms of sensitivity, specificity, positive predictive value, and negative predictive value no significant relation was observed.
